# Automated analysis of color tissue Doppler velocity recordings of the fetal myocardium using a new algorithm

**DOI:** 10.1186/s12947-015-0034-3

**Published:** 2015-08-27

**Authors:** Lotta Herling, Jonas Johnson, Kjerstin Ferm-Widlund, Peter Lindgren, Ganesh Acharya, Magnus Westgren

**Affiliations:** Centre for Fetal Medicine, Department of Obstetrics and Gynecology, Karolinska University Hospital, Stockholm, Sweden; University Hospital of Northern Norway, Tromsø, Norway; Department of Clinical Science, Intervention and Technology - CLINTEC, Karolinska Institute, Stockholm, Sweden; Department of Medical Engineering School of Technology and Health, KTH Royal Institute of Technology, Stockholm, Sweden; Department of Clinical Medicine, UiT- The Arctic University of Norway, Tromsø, Norway

**Keywords:** Tissue Doppler imaging, Fetal cardiac function, Region of interest (ROI), Automated algorithm

## Abstract

**Background:**

Tissue Doppler imaging (TDI) can be used to assess fetal cardiac function and it has been shown to detect changes associated with hypoxia in animal models. However, the analysis is cumbersome and time consuming. The main objective of this study was to evaluate the feasibility of a new algorithm developed for the automated analysis of color TDI velocity recordings of the fetal myocardium. Furthermore, we wanted to assess the effect of different sizes of region of interests (ROI) on the measurement of cardiac cycle time intervals and myocardial velocities at different gestations.

**Methods:**

This study included analysis of 261 TDI velocity traces obtained from 17 fetal echocardiographic examinations performed longitudinally on five pregnant women. Cine-loops of fetal cardiac four chamber view were recorded with color overlay in TDI mode and stored for off-line analysis. ROIs of different sizes were placed at the level of the atrioventricular plane in the septum and in the right and left ventricular walls of the fetal heart. An automated algorithm was then used for the analysis of velocity traces.

**Results:**

Out of the total 261 velocity traces, it was possible to analyze 203 (78 %) traces with the automated algorithm. It was possible to analyze 93 % (81/87) of traces recorded from the right ventricular wall, 82 % (71/87) from the left ventricular wall and 59 % (51/87) from the septum. There was a trend towards decreasing myocardial velocities with increasing ROI length. However, the cardiac cycle time intervals were similar irrespective of which ROI size was used.

**Conclusions:**

An automated analysis of color TDI fetal myocardial velocity traces seems feasible, especially for measuring cardiac cycle time intervals, and has the potential for clinical application.

## Background

Tissue Doppler imaging (TDI) is a technique that is used to evaluate the movements of the myocardial walls. In adults, it appears to facilitate the diagnosis of subclinical myocardial disease and helps in predicting the prognosis of major cardiac diseases, such as acute myocardial infarction [1–5]. The method has proven to be feasible in human fetuses [6, 7], and it has been tried for evaluating fetal cardiac function in various pregnancy complications, such as intrauterine growth restriction [8, 9], twin-to-twin transfusion syndrome [10] and gestational diabetes [11].

Studies on adults during hypoxia have found prolongation of ventricular isovolumic relaxation time measured by TDI [12]. This concurs with studies on sheep fetuses indicating that hypoxemia/acidemia is associated with prolonged flow Doppler isovolumic contraction time [13]. In an experimental fetal sheep model, prolongation of the pre- and post-ejection phases of the cardiac cycle during acute hypoxia/acidemia was demonstrated using color TDI [14]. The analysis of color TDI velocity traces could hypothetically give early indications of fetal hypoxia, as myocardial dysfunction is often the consequence as the circulation tries to adapt to a diminished oxygen supply.

There are two main echocardiographic approaches to assess myocardial motion, i.e. spectral (pulsed wave) TDI and color TDI. In both techniques, the region of interest (ROI) is generally placed at the level of the atrioventricular plane (AV-plane) in order to assess the longitudinal motion executed by subendocardial longitudinal myocardial fibers that are considered to be most susceptible to hypoxia [15, 16]. High resolution imaging is required to detect subtle changes in myocardial motion during different phases of the cardiac cycle. Since color TDI does not use fast Fourier transformation and can display velocity information in real time, it is plausible to think that it should have a higher temporal resolution than the spectral TDI [17]. Therefore, we hypothesized that color TDI is preferable for detecting changes associated with fetal hypoxia. However, acquisition and interpretation of color TDI in fetuses is associated with challenges related to fetal movements, high fetal heart rate and problems of defining time events in the cardiac cycle without a simultaneous ECG. Furthermore, the analysis is cumbersome and time consuming. The analysis includes image acquisition, transfer into other systems followed by visual identification and manual definition of cardiac cycle time events before quantitative information can be obtained. Since noninvasive recording of fetal ECG of adequate quality is not readily available, we designed a new method that enhances the information regarding myocardial velocity change over time. This allows for an automated analysis of myocardial velocities and a definition of cardiac cycle time intervals without concurrent ECG. This automated method of assessing cardiac cycle time intervals could potentially facilitate the application of color TDI as a tool for assessing fetal cardiac function.

To perform the automated analysis with this new method, well-defined velocity traces are required. Acceleration traces with distinct shifts that represent the changes in myocardial work can then be derived and used to define time intervals. The resolution of traces might be influenced by several factors including the size of the ROI, frame rate, size of the color box, sector width and line density. A larger ROI size has been shown to reduce the variability and diagnostic inconsistency in adults with dyssynchrony [18] and a smaller ROI size had a higher variance in measured parameters in a study on cats [19]. Using a larger ROI is expected to have a smoothing effect and reduce the measured velocities. However, as the main intention is to develop a rapid simplified method to assess changes in cardiac cycle time intervals, such as pre- and post-ejection phases that could potentially indicate fetal hypoxemia/academia, a loss of velocity information might be acceptable.

Clinical application of TDI for assessing and monitoring fetal wellbeing could be simplified and improved by automation of image analysis. Thus, the main objective of this study was to evaluate the feasibility of a new algorithm developed for the automated analysis of color TDI velocity recordings of the fetal myocardium. Furthermore, we wanted to assess the effect of different ROI sizes on the measurement of cardiac cycle time intervals and myocardial velocities at different gestations.

## Methods

This study included analysis of 261 TDI velocity traces obtained from 17 fetal echocardiographic examinations performed on five pregnant women with uncomplicated singleton pregnancies. These five women were randomly selected from a group of 120 women with uncomplicated pregnancies included in a larger longitudinal study investigating fetal cardiac function during 18–40 weeks of gestation. Ultrasound examinations were performed serially at approximately 4-weekly intervals by an experienced operator using a Vivid 7 Dimension ultrasound machine equipped with a M4S sector transducer with frequencies of 1.5–4.3 MHz (GE Vingmed Ultrasound AS, Horten, Norway.) Pregnancies were dated based on the measurement of biparietal diameter in the second trimester. The study was approved by the Regional Committee for Medical and Health Research Ethics – REK Nord (Ref.nr. 105/2008. Date of approval: 16.12.2008) and an informed written consent was obtained from all the participants.

**Fig. 1:**
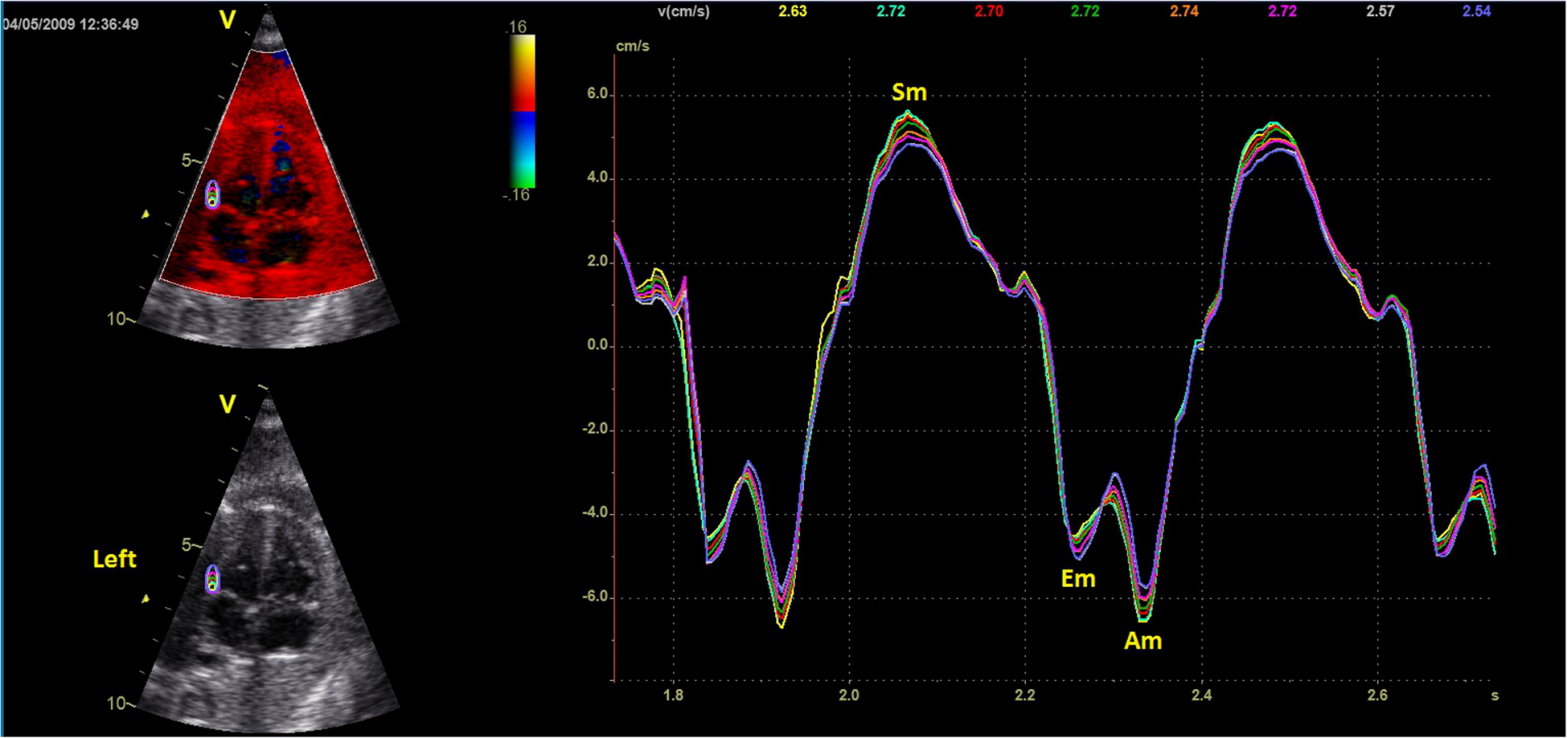
The position of region of interest (ROI). The left panel indicates position of the eight different ROI sizes in gestational age group III in the left ventricular wall. Different colors indicate different ROI sizes (height **×** width). Yellow (2 × 2 mm), turquoise (3 × 3 mm), red (4 × 3 mm), green (4 × 4 mm), orange (6 × 3 mm), pink (6 × 4 mm), grey (8 × 3 mm) and blue (8 × 4 mm). The right panel shows the velocity traces produced. Sm = peak systolic myocardial velocity. Em = peak early diastolic myocardial velocity. Am = peak myocardial velocity during atrial contraction

At each examination, a four chamber view of the fetal heart with color overlay in TDI mode was obtained and cine loops of 5-10 consecutive cardiac cycles were recorded. The ultrasound beam was aligned parallel to the interventricular septum with an insonation angle close to zero (<10°). An attempt was made to keep the frame rate >180 frames/s. An off-line analysis was performed using quantitative analysis (Q-analysis) in EchoPAC (GE Vingmed Ultrasound AS, Horten, Norway.) Fixed ROIs of different sizes were placed at the level of the AV-plane in the septum and in the right and left ventricular wall of the fetal heart. ROIs were placed manually at the level of the AV-plane (Fig. 1). The width of the ROI was increased in order to fit the increasing thickness of the septum or the ventricular walls.

The material was divided into three gestational age (GA) groups: GA I (18-24 weeks), GA II (25–32 weeks) and GA III (33–41 weeks). The largest ROI widths of 2 mm, 3 mm and 4 mm were chosen for GA I, GA II and GA III, respectively as estimated to cover the entire thickness of the myocardium. The ROI length was increased in steps to cover different proportions of the ventricular length. The length of the septum was measured from the AV-plane to the apex in end-diastole and ROI sizes compared to this as an estimate of proportion of myocardial ventricular length occupied by each ROI size. The largest ROI length in this study was 4 mm, 6 mm and 8 mm in GA I, GA II and GA III respectively. A ROI of 2×2 mm was analyzed for all recordings to enable comparison of myocardial velocities between different ROI sizes.

**Fig. 2:**
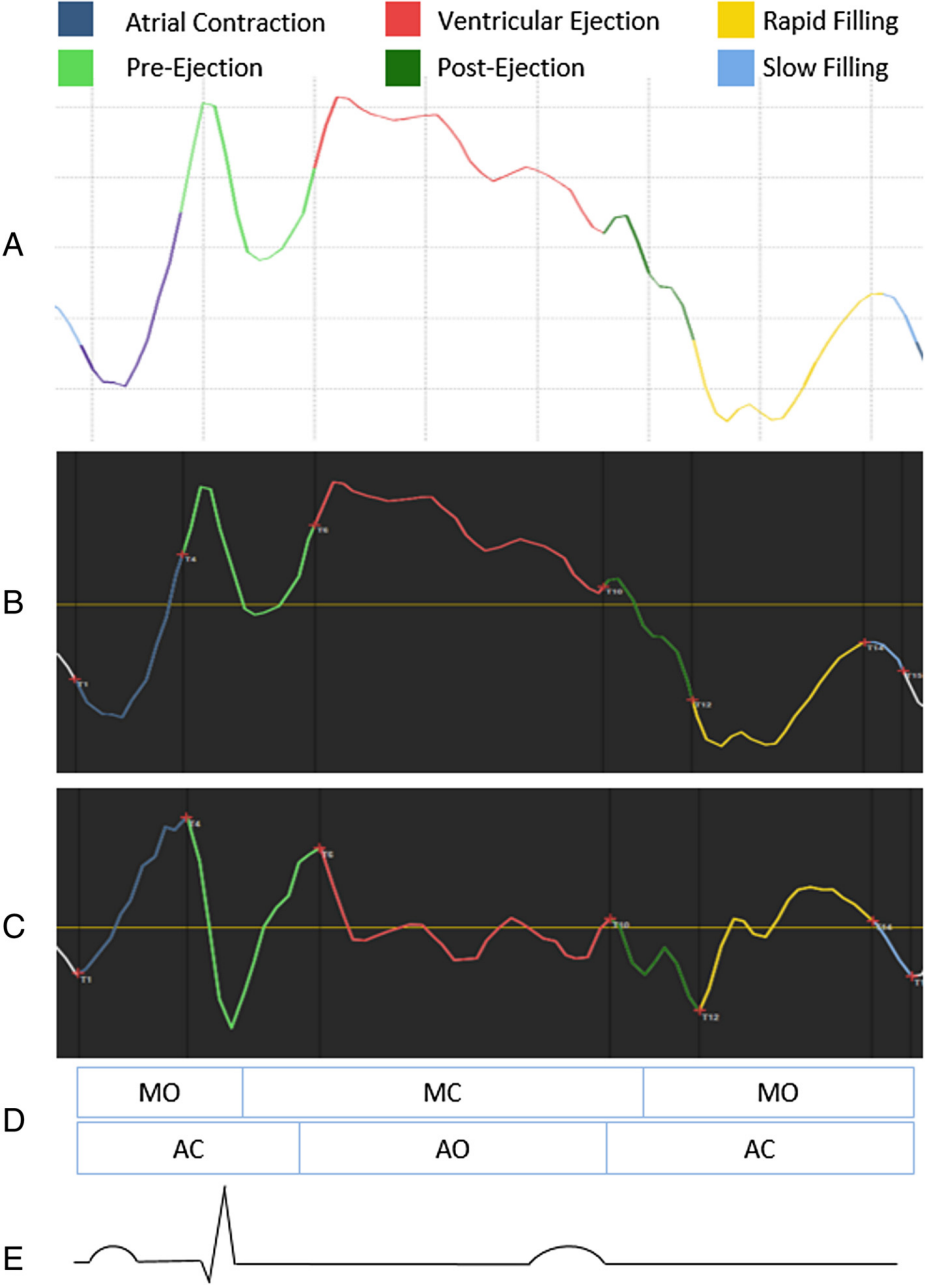
Definition of cardiac cycle time intervals. The different cardiac cycle time intervals are displayed at the top. **a** A myocardial velocity trace analyzed with the automated algorithm. **b** A myocardial velocity trace analyzed manually. **c** An acceleration trace where shifts have been identified manually in order to define cardiac cycle time intervals. **d** Estimated opening and closure of mitral valve (MO/MC) and aortic valve (AO/AC). **e** Estimated ECG reading indicating the likely position of P, Q, R and T wave

The myocardial velocity traces were transferred to GHLab software (Gripping Heart AB, Stockholm, Sweden) for visual identification and manual definition of cardiac cycle time intervals [20–22]. GHLab is a software that utilizes shifts in acceleration to define time events according to the *Dynamic Adaptive Piston Pump (DAPP)* principle describing the heart as a mechanical pump controlled by its inflow [23]. This method allows for identification of time intervals without a concurrent ECG signal. One representative cardiac cycle was chosen for the manual definition of acceleration shifts, subsequently generating six phases of the cardiac cycle: atrial contraction, pre-ejection, ventricular ejection, post-ejection, rapid filling and slow filling (Fig. 2). As the heart function, according to the *DAPP* principle, is initiated by the movement of the AV-piston (the AV-plane), the atrial contraction is considered as the starting point of the cardiac cycle. The terms pre- and post-ejection are used instead of isovolumic contraction and relaxation as they are defined by shifts in myocardial work rather than by the valve opening and closure.

**Fig. 3:**
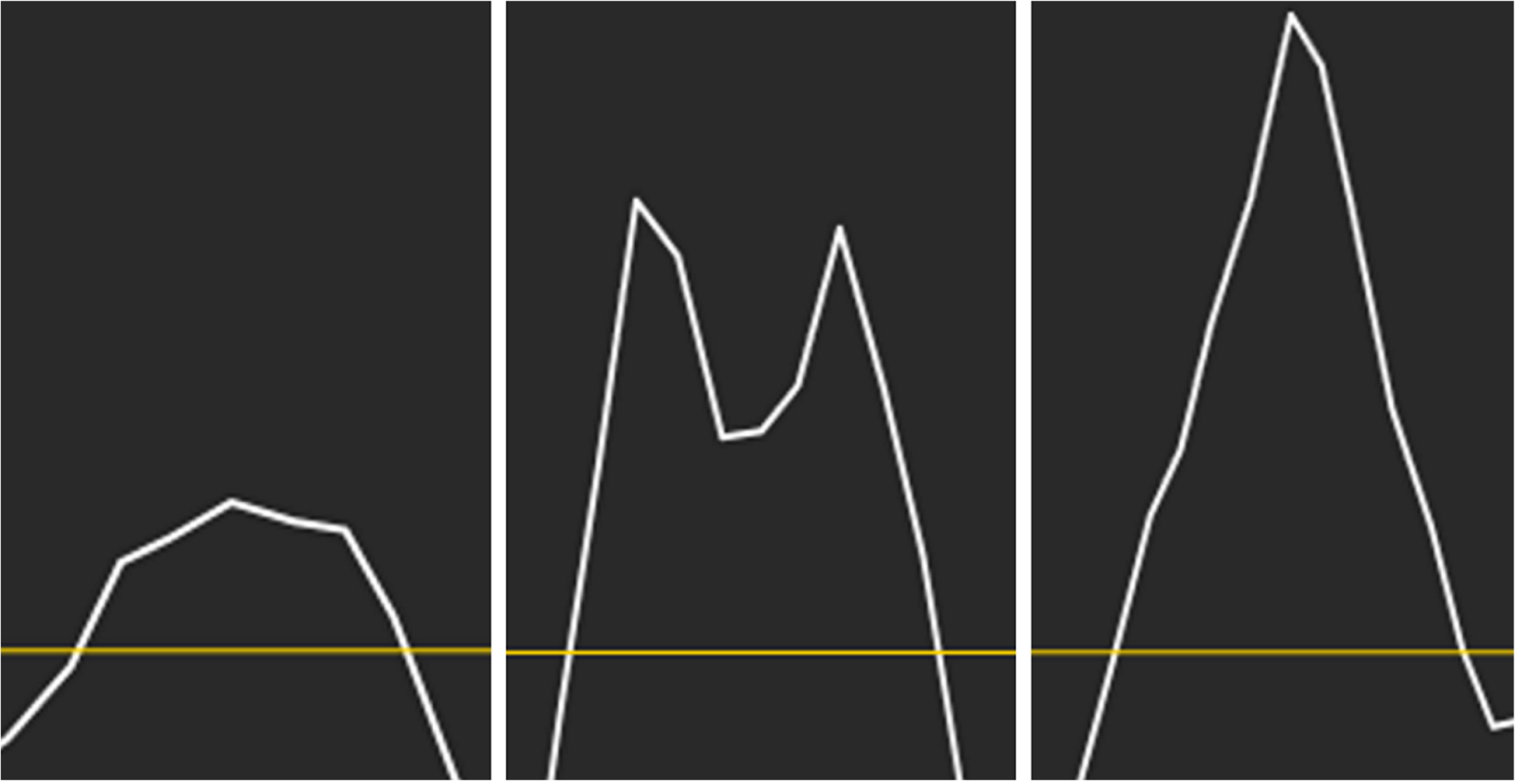
Typical acceleration traces. The left panel shows a trace with an acceleration score of 1, the middle panel with an acceleration score of 2 and the right panel with an acceleration score of 3

The acceleration shifts were assessed by two investigators (LH and JJ) and the traces were divided into three categories depending on the degree of well-defined shifts. An acceleration score of 3 represents clear and well-defined shifts, a score of 2 less well-defined, often with a biphasic appearance, and a score of 1 with indistinct shifts, generally with a flat appearance (Fig. 3). An average score was calculated for the septum, right and left ventricular wall. The total number of optimal traces with an acceleration score of 3 for each ROI size was recorded as a measure of quality.

Information regarding peak myocardial velocities during early diastole (Em), atrial contraction (Am) and systole (Sm) were automatically extracted from the traces by the GHLab software as soon as the time intervals were defined. All measured velocity parameters were compared to the 2×2 mm ROI as the smallest ROI size is expected to record the highest velocities being closest to the AV-plane and averaging a smaller area. The differences in velocities and time intervals measured using different ROIs were calculated as the percent change in relation to the reference ROI of 2×2 mm.

A newly developed automatic algorithm was then used to analyze the original TDI velocity traces and obtain cardiac cycle time intervals using MATLAB (R2010a, MathWorks, MA, USA). The automated algorithm consists of approximately 100 pages of MATLAB code, encompasses four different filter settings and uses pattern recognition to detect time events.

In order to assess limitations, extended information was extracted from the DICOM files. For the evaluation of axial resolution the points/mm along the ultrasound beam was measured, for temporal resolution the frame rate and for the spatial resolution the line density was noted. The traces were smoothed by a three point moving average filter in EchoPAC.

Data analysis was performed using IBM SPSS Statistics for Windows, version 23.0 (IBM Corp., Armonk, N.Y., USA). Continuous variables are presented as mean ± SD or median (range) as appropriate. Categorical variables are presented as (n %).

## Results

The mean age of the pregnant women was 29 ± 4.4 years, BMI was 23.7 ± 2.6 kg/m^2^ and all women had uncomplicated pregnancies with normal perinatal outcome. The median gestational age at examination was 21 weeks in GA I, 29 weeks in GA II and 38 weeks in GA III. The total number of analyzed traces including all walls and ROI sizes were 45, 72 and 144 respectively. The axial resolution of images estimated by the median number of points/mm was 1.46 (range, 1.31–1.49), the median frame rate (temporal resolution) was 190 (range, 179–219) per second, and the line density (spatial resolution) was 20 (range, 20–28). The median height of the color box during the examination was 10.8 cm (7.2–12.7 cm).

The different ROI sizes used in each gestational age group, the total number of traces with an acceleration score of 3, mean ventricular length of the septum and percentage of the ventricular length occupied by each ROI size are presented in Table 1. The number of traces with an acceleration score of 3 increased with ROI length in GA II and GA III but not in GA I. All velocity traces were possible to analyze with the manual method to identify and measure the cardiac cycle time intervals. Out of the total 261 traces, it was possible to analyze 203 (78 %) of traces with the automated algorithm. It was possible to analyze 93 % (81/87) of traces recorded from the right ventricular wall, 82 % (71/87) from the left ventricular wall and 59 % (51/87) from the septum. The average acceleration score was 2.80 for the left ventricular wall, 2.67 for the right ventricular wall and 2.51 for the septum. The automated analysis with three exported velocity traces required <1 s for analysis compared to the manual method that required approximately 2–3 min.

**Table 1:** Region of Interest (ROI) sizes

Gestational age group	ROI size (mm)	Acceleration score 3 traces (n)	Length of the septum (mm)	ROI length
GA I (18–24 weeks)	2 × 2	6		13 %
	3 × 2	6	15.6 ± 2.3	19 %
	4 × 2	5		26 %
GA II (25–32 weeks)	2 × 2	5		9 %
	3 × 3	8	23.3 ± 2.6	13 %
	4 × 3	10		17 %
	6 × 3	11		26 %
GA III (33–41 weeks)	2 × 2	4		6 %
	3 × 3	5		9 %
	4 × 3	5		12 %
	4 × 4	7	32.5 ± 2.0	12 %
	6 × 3	9		18 %
	6 × 4	9		18 %
	8 × 3	11		25 %
	8 × 4	12		25 %

ROI size is presented as height × width, length of the septum as mean ± SD and ROI length as % of septal length

**Fig. 4:**
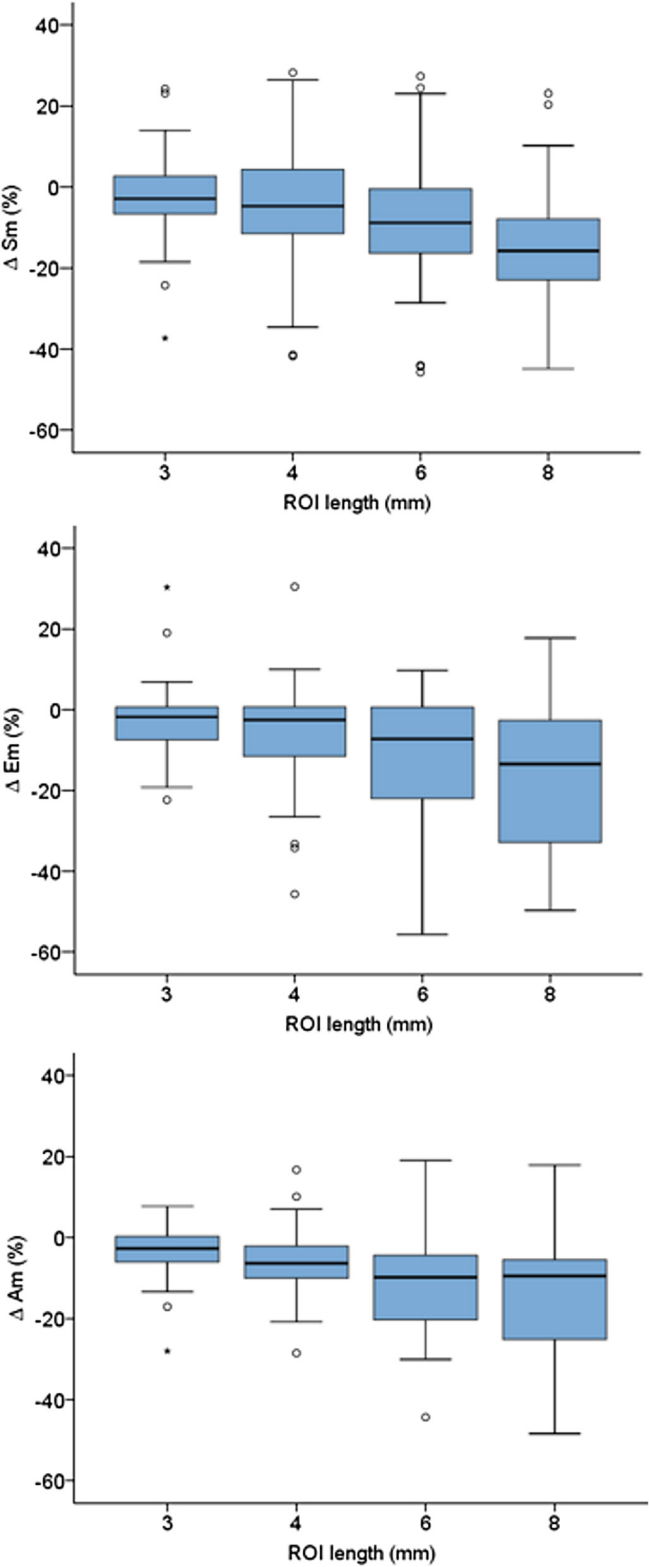
Difference in peak myocardial velocities between different ROI sizes. Sm = peak systolic myocardial velocity. Em = peak early diastolic myocardial velocity. Am = peak myocardial velocity during atrial contraction. ROI – region of interest

The percent differences in velocities measured using different ROI lengths compared to the reference ROI of 2×2 are shown in Table 2 and Fig. 4. There was a clear trend towards decreasing myocardial velocities with increasing ROI length. The interquartile range also increased substantially with increasing ROI length. However, the cardiac cycle time intervals showed minimal variation irrespective of which ROI size was used (Table 3).

**Table 2:** Difference in peak myocardial velocities between different ROI lengths according to gestational age (GA) group

	ROI length (mm)			
	3	4	6	8
Δ Sm (%)				
GA I	−3.49 (14)	−9.27 (19)		
GA II	−1.57 (7)	−1.04 (11)	−8.19 (16)	
GA III	−2.86 (9)	−4.60 (17)	−9.24 (16)	−15.79 (16)
Δ Em (%)				
GA I	−1.18 (9)	−6.27 (10)		
GA II	−0.11 (7)	−0.70 (11)	−1.93 (20)	
GA III	−3.16 (14)	−5.98 (18)	−8.46 (27)	−13.49 (31)
Δ Am (%)				
GA I	−2.34 (6)	−8.62 (9)		
GA II	−4.78 (8)	−8.71 (16)	−13.66 (16)	
GA III	−2.11 (6)	−5.22 (6)	−7.74 (14)	−9.52 (20)

Data are presented as median difference (Δ) % (interquartile range) compared to the reference ROI of 2×2 mm. ROI = region of interest. Sm = peak systolic myocardial velocity. Em = peak early diastolic myocardial velocity. Am = peak myocardial velocity during atrial contraction. GA I – gestational age group I. GA II – gestational age group II. GA III – gestational age group III

**Table 3:** Difference in percentage for time intervals between different ROI lengths compared to the reference ROI

	ROI length (mm)	Atrial contraction	Pre-ejection	Ventricular ejection	Post-ejection
GA I	3	−1.04 (5)	0.92 (5)	0.00 (2)	1.94 (7)
	4	−3.18 (5)	0.59 (9)	0.00 (3)	1.55 (7)
GA II	3	0.00 (6)	0.00 (4)	0.00 (2)	0.88 (7)
	4	1.13 (8)	1.25 (4)	0.26 (2)	0.54 (8)
	6	−1.43 (7)	0.97 (6)	−0.30 (2)	1.97 (9)
GA III	3	−1.75 (7)	1.09 (8)	−0.02 (4)	1.69 (4)
	4	−0.06 (6)	−0.21 (9)	0.10 (4)	1.83 (6)
	6	0.07 (11)	0.67 (10)	0.14 (4)	2.59 (8)
	8	−1.09 (9)	0.71 (13)	0.20 (3)	2.57 (6)

Data are presented as median difference (Δ) % (interquartile range) compared to the reference ROI of 2×2 mm. ROI = region of interest. GA I – gestational age group I, GA II – gestational age group II, GA III – gestational age group III

## Discussion

Functional fetal echocardiography using TDI is a promising tool in experienced hands for a selected group of patients. However, its introduction to more general clinical practice is hampered partly because it is cumbersome and time consuming. Our study indicates that automated analysis of fetal cardiac function assessed by color TDI is feasible using a newly developed algorithm. If the automated method could be used for instantaneous on-line display of results it could prove to be clinically useful. The automated technique requires substantially less time to analyze the TDI velocity traces compared to the manual method. The method is not dependent on an ECG in order to define time intervals and therefore suitable for antenatal assessment of human fetuses. As the automated method uses enhanced acceleration shifts rather than crossing points of velocity traces with the baseline for defining different phases of the cardiac cycle, it can be expected to reduce the errors related to fetal breathing and movements. Furthermore, multiple cardiac cycles can be evaluated quickly and averaged, which could result in a potentially higher accuracy of measurements compared to a manual analysis.

The automated method appears to be dependent on the quality of velocity traces and its derivatives, i.e. acceleration traces, and these seem to vary with the size of the ROI. The fetal heart grows considerably throughout gestation. Tan et al. have measured the thickness of the septum in a fetal four chamber view at end-diastole giving wall dimensions of 1.8 mm, 2.9 mm and 3.6 mm at a gestational age of 20, 30 and 40 weeks respectively. Studies of the right ventricular cavity at end-diastole demonstrate the length to be 12 mm, 20 mm and 26 mm at 20, 30 and 40 weeks respectively [24, 25]. As the fetal heart grows with advancing gestation we would suggest increasing ROI sizes with increasing gestational age. The width of the ROI should cover the width of the wall measured and, therefore, it seems adequate to choose 2 mm in GA I, 3 mm in GA II and 4 mm in GA III. While choosing ROI length the one producing distinct acceleration traces should be selected in order to optimize the automated method. However, an excessive loss of velocity information should be avoided. Therefore we suggest the use of a 2×2 mm ROI in GA I (18–24 weeks), a 4×3 mm ROI in GA II (25–32 weeks) and a 6×4 mm ROI in GA III (33–41 weeks).

TDI might be a useful method in assessing fetal cardiac function, but there is a lack of standardization concerning image acquisition, data analysis and post-processing. As we use a fixed (stationary) ROI placed at the level of the AV-plane at end systole, the myocardium apical to the original position will be interrogated during the rest of the cardiac cycle. Therefore, it might be important to choose a reasonably large ROI to reduce the variability associated with operator placement of the ROI [18]. Substantial loss of velocity information and increasing interquartile ranges with increasing ROI length are limitations that should be considered while assessing myocardial velocities. The main focus of this automated method was, however, the assessment of cardiac cycle time intervals, and a larger ROI size seems to optimize traces for this purpose and consequently could improve automation.

Although only a small number of women with normal pregnancies were examined, for a feasibility study we had a reasonable number of traces (*n* = 261) to evaluate. However, all color TDI recordings are unlikely to be adequate for evaluation even by manual method in a larger unselected population. The accuracy of the automated algorithm should be tested in a larger population including pathological pregnancies.

## Conclusions

An automated analysis of color TDI fetal myocardial velocity traces seems feasible, especially for measuring cardiac cycle time intervals, and has the potential for clinical application.
